# Legal Notes for Institutional Workers

**Published:** 1915-08-21

**Authors:** 


### LEGAL NOTES FOR INSTITUTIONAL WORKERS.

### Institutional Friction.

An Irish case, *Mayne* v. *County Council of Longford*, tried by Mr. Justice Gibson at the Longford Assizes last month, illustrates yet again the confusion and difficulty which inevitably ensue when local authorities and their officials do not act in harmony. The plaintiff was the surgeon of the county infirmary, which institution was administered by a committee of the Council. A controversy arose in consequence of the refusal of the surgeon to allow another surgeon to perform an operation on a soldier in the infirmary. In so acting the plaintiff was strictly correct, but by way of getting even with him, resort was had to the statute 6 and 7 Will. 4, c. 116, s. 86, under which the surgeon cannot get paid his salary unless five members of the committee sign a certificate stating that certain conditions have been fulfilled. Four persons signed the document at a duly convened meeting; and one person otherwise qualified signed it but was not present at the meeting. The Council refused to pay, and the surgeon sued in the County Court for the amount of salary due. The County Court judge gave decree, but Mr. Justice Gibson said that it was impossible to get over the objection raised as to the irregularity of the certificate founded on; and he reversed the decision of the inferior judge; adding that, though it was not his place to advise the plaintiff, he thought his proper remedy was to apply in the King's Bench for a *mandamus* to the infirmary governors to do the acts necessary to secure payment to him of the moneys due.

### Heavy Damages for a Doctor.

We notice from the Press two cases which, if they contain no point of legal or medico-legal principle, may yet be noticed for this: that in both of them the character and repute of the medical practitioner involved was justified. In one of them a Winchelsea surgeon was summoned for alleged indecent assault on a patient, but which the Bench refused to send to trial, the chairman stating that the defendant left the court without the slightest imputation on his moral or professional character; a remark was, however, made to the effect that the doctor was unwise in conducting an examination of the kind to which the patient had, of her own consent, been subjected, without the presence or near proximity of a third person.

The other case was a slander action arising out of differences as to parish nursing between the medical officer of Haddington Parish and a lady who was president of the Haddington Parish Nursing Association. The alleged slander was contained in a letter addressed by the defender to the chairman of the Parish Council of Haddington, which, the defender averred, falsely and calumniously represented that he had been guilty of gross and wilful failure to discharge his duty as a professional man by omitting to procure for one of his patients "the help which was within reach and to which she was entitled," and which letter also contained the following passage: "It is almost incredible that persons unfortunate enough to be on the roll should be allowed to suffer and die without being given the services of a trained nurse, whose services were being actually paid for by a grant out of the rates." As is not uncommon in those cases, the evidence wandered over a wide field, and embraced a great deal of irrelevant matter. The drift of it was plainly this, that while the defender was anxious to give sick people the benefit of a trained nurse on all occasions of illness, the pursuer's view was that in difficult cases where, for instance, dressings had to be done, it would be advantageous to have the trained nurse in, but in simple cases he thought the patient would be as well, if not better, attended to by a neighbour. The presiding judge directed the jury to what he considered the sole question in the case—namely, what was the meaning of the letter?—and he gave them the firm direction in law that there could be no case of privilege on the part of the defender, for he had, on the evidence, reached the conclusion that the patient who was in question was being treated by the doctor as a private patient, and had nothing to do with the Parish Council and the Parish Council had nothing to do with her, so that in writing the chairman of the Parish Council as she did, the defender could not claim that she was in the privileged position of one entitled to animadvert on a matter of public interest. The jury returned a verdict for the pursuer and assessed the damages at £1,000. If we may express an opinion, it does seem to us a pity that a case like this should go to such a length. The defender was acknowledged to be a public-spirited woman who had only the interests of the sick poor at heart; and there was no suggestion of professional incompetence made against the pursuer. All the trouble was apparently due to a difference in policy and opinion between two strong-willed personalities; and it is regrettable that some mediating and reconciling influence had not interposed.

